# Environmental Earth Sciences Progress Report 2020 and Outlook 2021

**DOI:** 10.1007/s12665-021-09531-8

**Published:** 2021-04-09

**Authors:** Olaf Kolditz, Paola Teti, Gunter Dörhöfer, Jim LaMoreaux, Gioacchino F. Andriani, Stephen Appleyard, Ted Asch, Gabriele Buttafuoco, Peter Dietrich, Andrew Hursthouse, Derek Kim, Broder J. Merkel, Jan Schwarzbauer, Siegfried Siegesmund, Barbara Kolditz

**Affiliations:** 1 Helmholtz Centre for Environmental Research UFZ / TU Dresden, Leipzig, Saxony Germany; 2 Springer Nature, Heidelberg, Germany; 3 EES Office Brandenburg, Brandenburg a.d.Havel, Germany; 4 EES Office Tuscaloosa, Tuscaloosa, Alabama USA; 5 Università degli Studi di Bari, Bari, Italy; 6 University of Western Australia, Perth, Australia; 7 Aqua Geo Frameworks, LLC, Lakewood, Colorado USA; 8 National Research Council of Italy, Rende, Italy; 9 Helmholtz Centre for Environmental Research UFZ / University of Tübingen, Leipzig, Saxony Germany; 10 University of the West of Scotland, Paisley, Scotland UK; 11 Water and Waste Management and Remediation, Fullertone, California USA; 12 TU Bergakademie Freiberg, Freiberg, Saxony Germany; 13 RWTH Aachen University, Aachen, Germany; 14 University of Göttingen, Göttingen, Germany; 15 EES Office Leipzig, Leipzig, Saxony Germany

**Keywords:** Environmental Earth Sciences (EES), Most cited papers, Progress report, 2020

## Abstract

The present editorial 2020 continues the series of status reports in Environmental Earth Sciences (EES) in previous years 2017 and 2019 (Kolditz et al. in Environ Earth Sci 77: 8, 2018, Kolditz et al. in Environ Earth Sci 79: 11, 2020). The year 2020 coming to an end was heavily influenced by the COVID-19 pandemic affecting all areas of life including research work and, therefore, scientific publishing as well (“Introduction”). One bright spot which shows longevity of journals that produce a quality product is that Environmental Earth Sciences (EES) is celebrating its 45th anniversary of publication. To this extent EES continues the tradition to honor the most cited papers contributing to the 2020 Impact Factor (IF) (“Highly and most cited topics”) and provide information on the current status of EES as well as an outlook to 2021 (“Progress report”)

## Introduction

Over the last year, the COVID-19 pandemic has caused significant economic and social effects throughout the world, and has caused significant impacts on the way that the research community has been able to live and work during this period. As a result of the pandemic, immense research efforts have been directed towards improving our understanding of the behaviour of the virus and of ways to defeat it. The way in which scientific communication has been changed entirely, as conferences are now mainly held in virtual formats. Video conferencing now allows information to be exchanged more frequently and quickly without travelling. However, the intensity and creativity when meeting and talking in person is missing, and our communication has become rather “two-dimensional” due to our reliance on connecting with each other through a computer screen. For environmental sciences, in particular the field work has become more difficult due to travel restrictions. In general, however, the publication success of EES has not been significantly impacted.

**Fig. 1:**
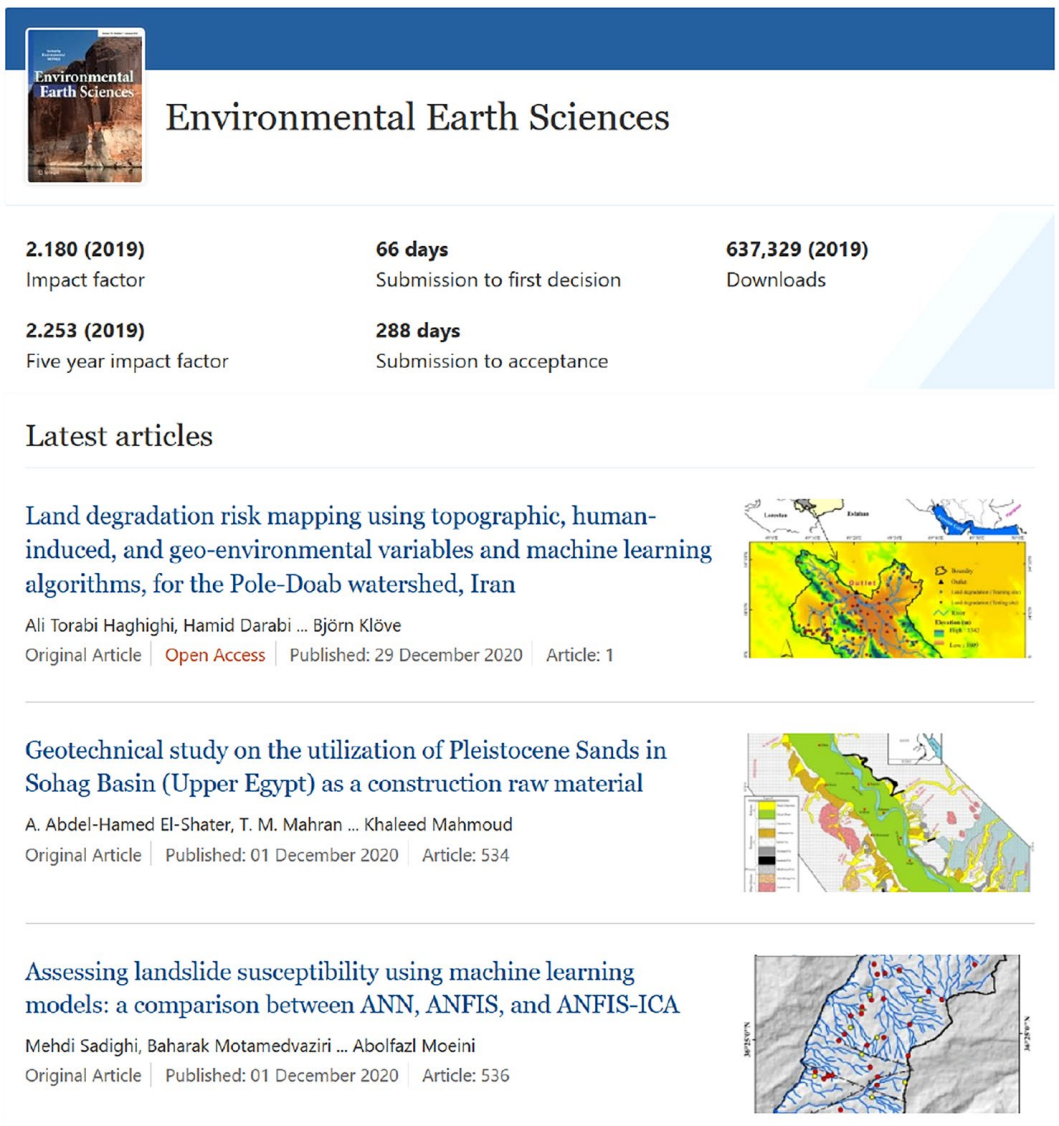
The door to EES: https://www.springer.com/journal/12665

In 2019, a new Publishing Editor, Paola Teti, was assigned by Springer and the editorial workflow was changed as well. Paola follows a distinguished group of former Publishing Editors: Drs. Wolfgang Engel, Christian Witschel, and Annett Buettner. Editors during this time were Drs. Peter Flawn and Philip LaMoreaux. Drs. Jim LaMoreaux and Gunter Dorhofer succeeded them and Olaf Kolditz is the most recent addition to the Editors-in-Chief of EES. These distinguished scientists have helped establish EES as a well-respected journal known for its applied approach to science with an emphasis on the coverage of research in developing countries^1^.

With our new Publishing Editor, Paola Teti, the editorial workflow has been changed as well. The Editors-in-Chief (EiCs), Managing Editors and Publishing Editor have initiated editorial meetings to discuss pressing topics and accelerate decision-making processes. The habits and technical progress of video meetings have been utilized to update the format of the Annual Editorial Meetings. The Associate Editors are invited to the annual meetings to share their expertise and experience on a regular basis.

During the journal editors meeting in November 2020, the discussion on the scope of the journal was continued. EES’s focus is on geoscientific topics and related environmental impacts—studies of real-world problems and developing solutions for them. The sustainable development goals (SDG) serve as a compass for the societal relevance of research and development in this field. To emphasise this direction, a journal theme for “Case/Field Studies” is currently being considered. The issue of how to further improve the quality and speed of manuscript handling is a permanent issue that is being considered by the Editors. Examples of how this could be done include shortening the duration of invitation request times for reviewers, and by updating the reviewers’ data base.

The EES website, which was launched in 2019, is now fully functional and allows readers to easily grasp contents and to attract the interest of readers. The title page contains useful information on basic statistics (impact factors, time from submission to first decision and acceptance, paper download). The latest articles from the journal are highlighted with a key figure or photo (Fig. 1). During 2020, 536 articles were published.

## Highly and most cited topics

**Fig. 2:**
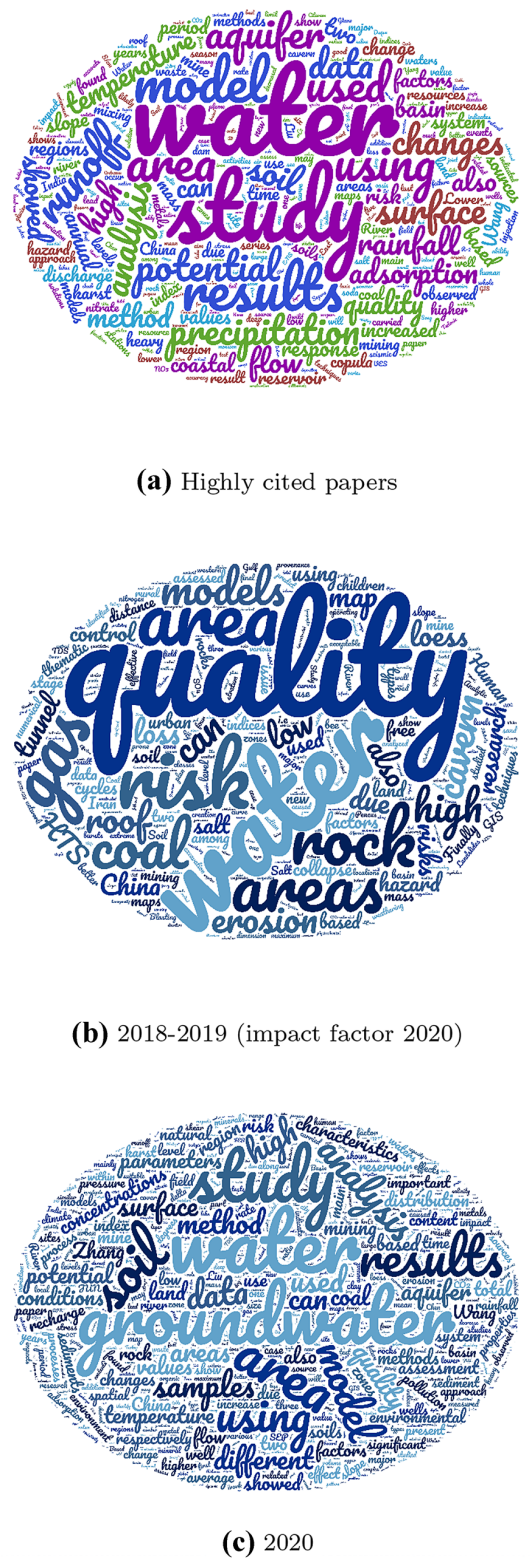
“Wordle” of the (**a**) highly cited, (**b**) most cited papers 2018–2019, and (**c**) 2020, respectively (using Wortwolken™) via http://www.wortwolken.com/

“Wordles” or “word clouds” have been used again to identify the most-used words in papers’ titles and abstracts. Even though not acknowledged as a rigorous scientific approach, it provides an interesting insight into the most frequently covered topics. The size of the words in the picture corresponds to their coverage in the paper titles and abstracts. Figure 2 illustrates three categories: (a) highly cited papers; (b) papers affecting the impact factor in 2020; and (c) topics in 2020:

- Highly cited papers: As per its definition, a highly cited paper received enough citations as of four  to  5 months ago to place it in the top 1% of its academic field based on a highly cited threshold for the field and publication year. Geosciences rank in 12th place in research fields of highly cited papers according to InCites Essential Science Indicators of Clarivate Analytics. EES currently (as of 31.12.2020) has 16 highly cited papers belonging to the top 1% cited in July/August 2020 in its research fields. Water-related topics belong to the most frequently cited category (Fig. 2a).
- 2018-2019: Water quality, risk, rock, and gas are the leading topics in most cited EES’ papers for the upcoming impact factor 2020 (Fig. 2b).
- 2020: Water, groundwater, and soil are the most utilized words in EES’ 2020 papers (Fig. 2c).

As a tradition of the EES editorial analysis, among the large number of excellent works, the most cited papers for the upcoming impact factor 2020 are highlighted here. These eight are highly cited as well (see above). This procedure corresponds to the calculation of a journal’s impact factor for 2020^2^. Please note that the present editorial does not influence the impact factor calculation and related self-citations as all 2020 papers are published already.

The Editors are pleased to award the most cited EES papers as follows: papers listed in the references include the number of citations in the ”Web-of-Science” (WoS)^3^ and in the SCOPUS data base^4^ (the citation count is as of 31.12.2020):

1. Zhang et al. (2018) (highly cited paper)
2. Wu et al. (2019) (highly cited paper)
3. Li et al. (2018)
4. Qin et al. (2019) (highly cited paper)
5. Lai et al. (2018)
6. Pham et al. (2018)
7. Skilodimou et al. (2019) (highly cited paper)
8. Arabameri et al. (2018)
9. Zhang et al. (2019) (highly cited paper)
10. Wang et al. (2019) (highly cited paper)
11. Lu et al. (2019) (highly cited paper)
12. Koopialipoor et al. (2019) (highly cited paper)
13. Li and Qian (2018)
14. Qasemi et al. (2018)
15. Huang et al. (2018)

Congratulations go not only to authors of these most cited papers but also to all EES authors and reviewers for their excellent contributions to the journal.

## Progress report

The journal’s progress report summarizes selected indicators and statistics concerning journal development in the impact-factor-related years 2018-2020. The impact factor is a key criterion concerning the scientific impact of the journal in the research field. It characterizes the quality of the journal and acceptance within the scientific community. More recently, however, the DORA Agreement has been signed which expands the ways to measure journal quality. In addition to providing a journal of high-quality scientific content, EES strives to provide a platform and broad forum for scientists all over the world by addressing diverse environmental issues and challenges particularly in developing countries.

**Fig. 3:**
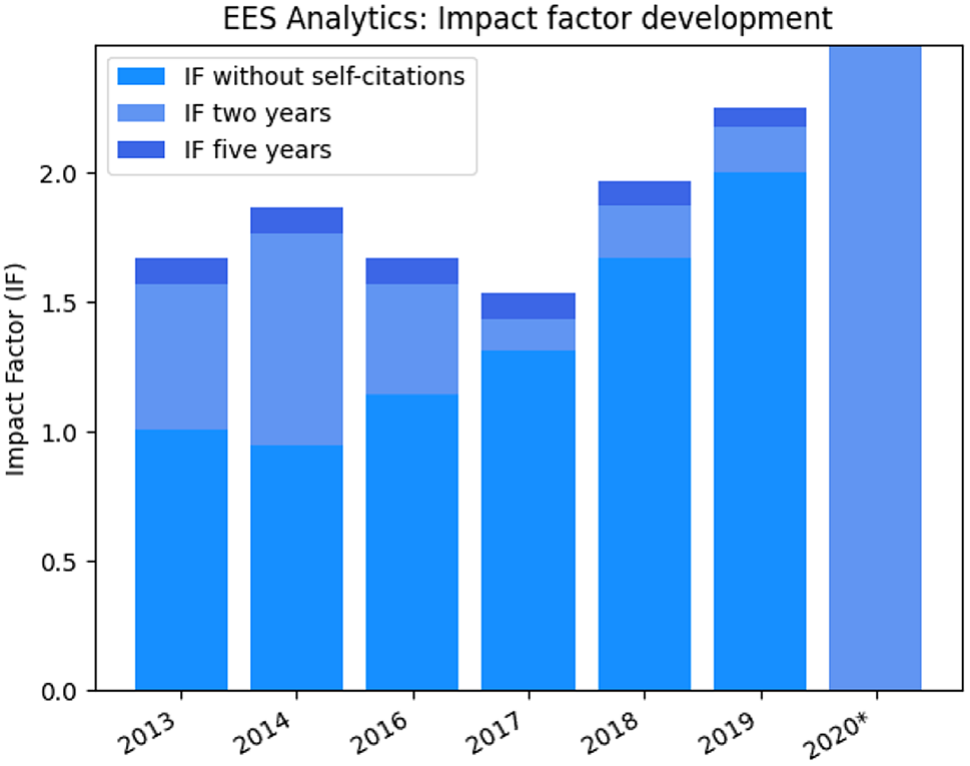
EES Impact Factor development (Source: Clarivate Analytics)

### Impact factor development

Figure 3 illustrates impact factor (IF) development since 2013^5^. Three categories are shown: the IFs for 5 and 2 years, respectively, as well as the 2-year impact factor without self-citations. The impact factor has increased continuously since 2017. The impact factor without self-citations has increased continuously since 2014, which underpins the increasing impact outside the journal itself. The overall self-citation percentage (over all years) is equal to 13.31%. In 2019 the IF exceeded the number 2 for the first time. The estimated impact factor 2020* is calculated based on data as of December 31, 2020^6^. All data for journal analysis come from Clarivate Analytics.

**Fig. 4:**
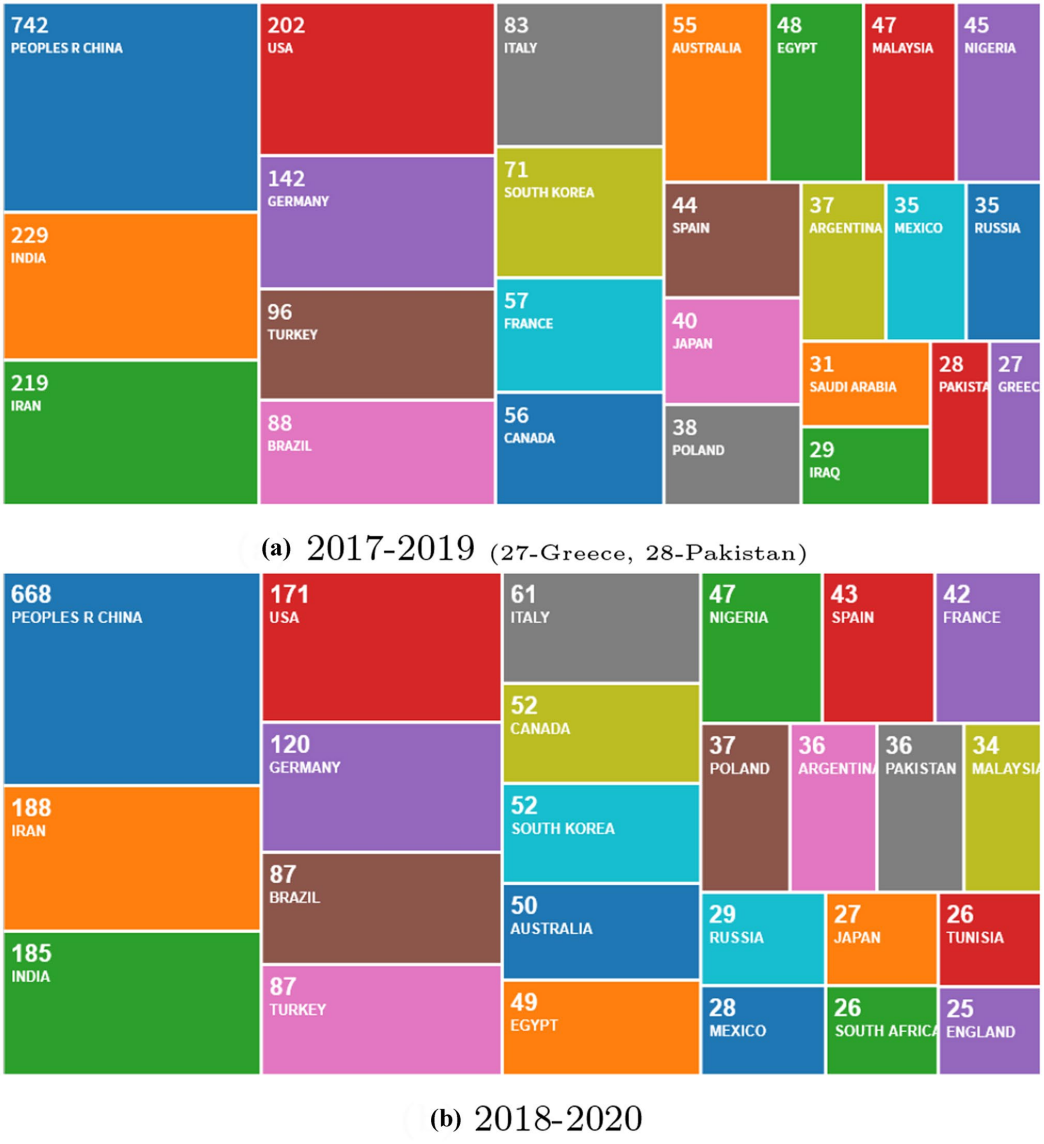
EES publications by countries (Source: Clarivate Analytics)

### Authors’ and institutional contributions

Authors’ contributions of publications by countries and by institutions from 2017 to 2019 and 2018 to 2020, respectively, are shown. Fig. 4 depicts the 25 most frequent citizenships of the corresponding authors. China is leading concerning published items, followed by a second group of authors from India, Iran, USA, and Germany with more than 100 publications. The second group with a similar portion provides about 60% of published items. India and Iran as well as Turkey and Brazil changed their positions in the first and second groups, respectively. An increasing number of publications from Tunisia, South Africa, and England have now appeared now in the top 25 list. Other initiatives dedicated to the preparation of Topical Collections in collaboration with guest editors are in the pipeline to broaden the geographical origin of contributing authors.

**Fig. 5:**
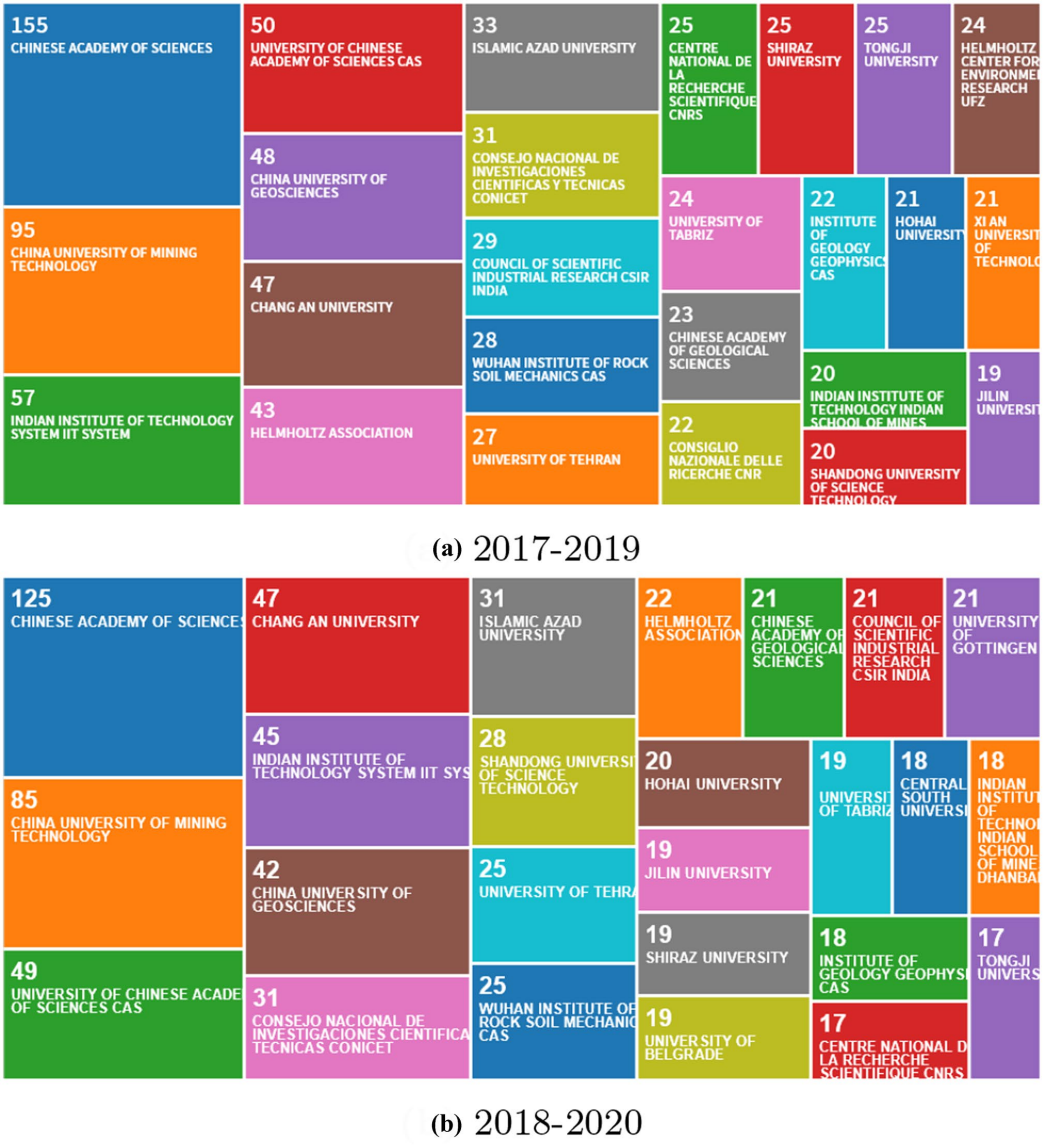
EES publications by affiliations (Source: Clarivate Analytics)

Figure 5 shows the analysis by research affiliations, national research institutions and universities. When comparing the data for the 2017-2019 and 2018-2020 periods, a small decline is observed of publication numbers but the composition of contributing institutions to the journal remains stable.

### Submissions and publishing

**Fig. 6:**
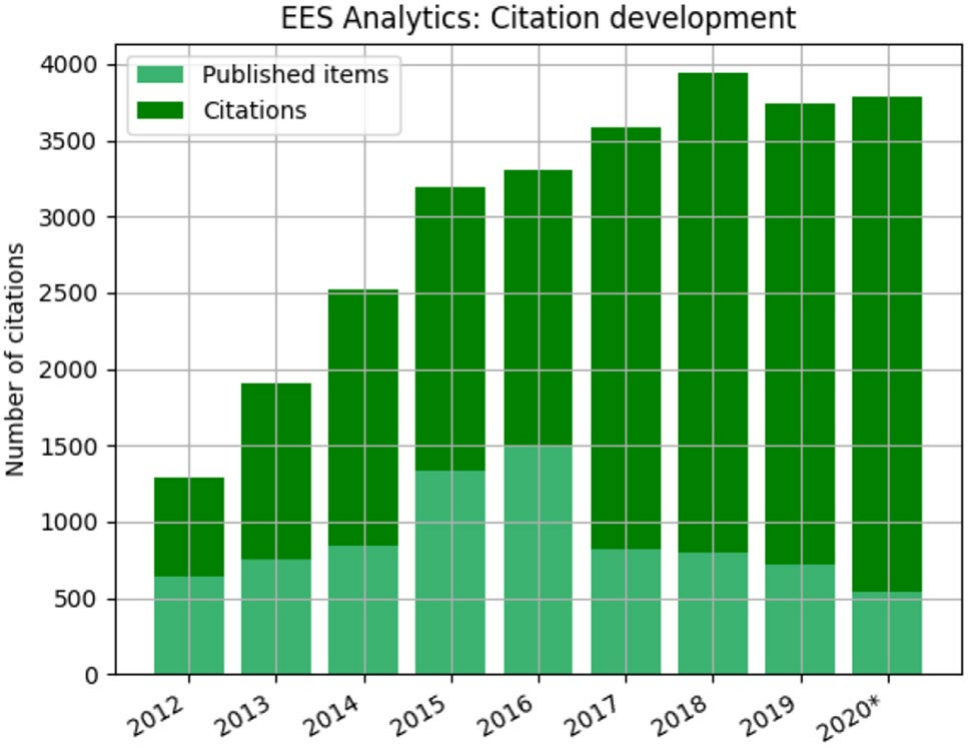
Citations versus published items (Source: Clarivate Analytics) [Status 31.12.2020, the number of citations for last year normally is still increasing until spring of the ongoing year]

**Fig. 7:**
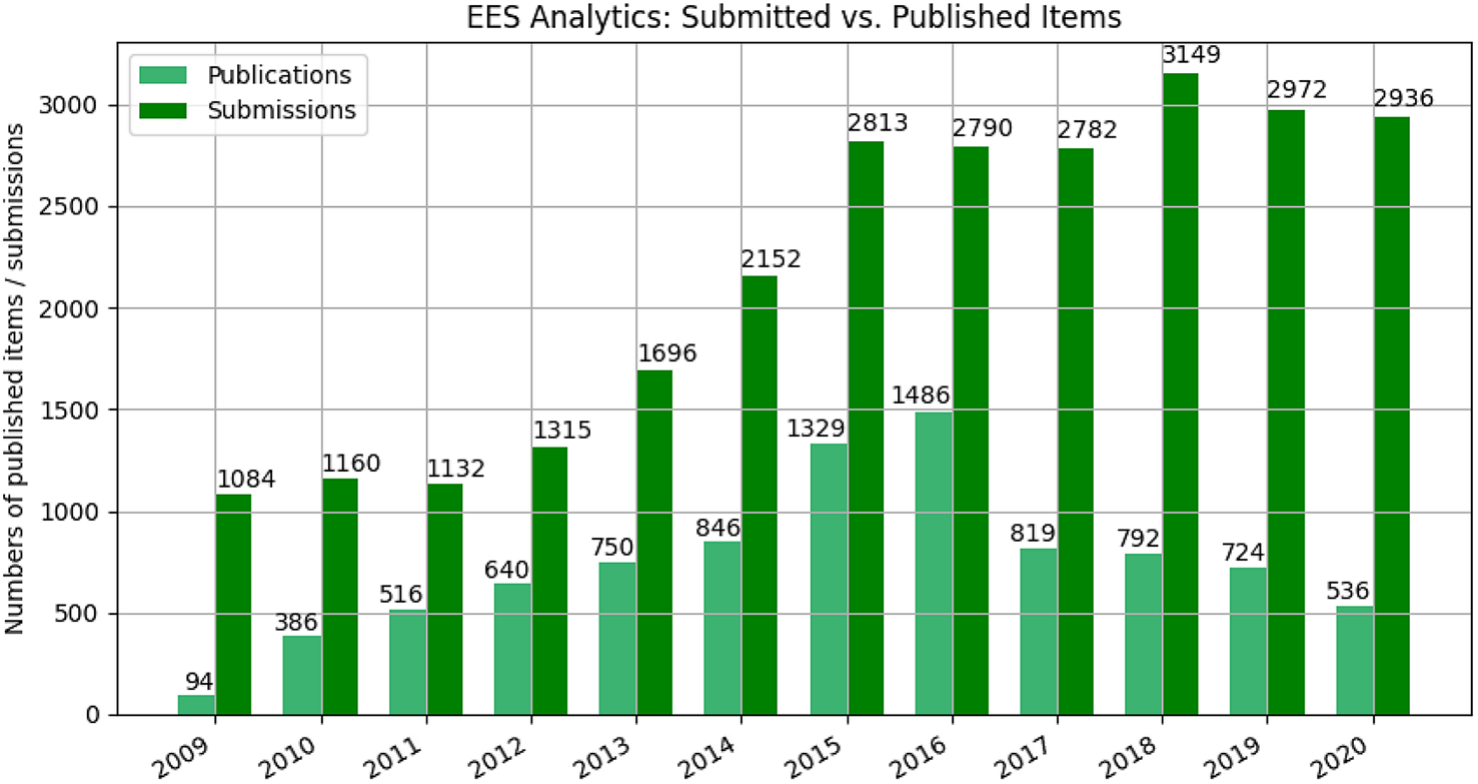
Submitted versus published items (Source: EES Editorial Manager)

Continuous article publishing (CAP) became the standard for processing articles in EES in 2016 and has positively affected the entire publication process. Currently it takes an average of 66 days from submission to first decision and 288 days to acceptance (Fig. 1)^7^. The relation between published articles and citations is illustrated in Fig. 6. Up to 2018 the number of citations increased to almost 4000 although currently it is experiencing a small decline^8^. The numbers for citations and published items have to be viewed in the context of impact factor development (cf. Fig. 3).

The final graphics show the relationship between submission and published items (Fig. 7). In 2018, the number of submissions exceeded 3000 for the first time and the number of submissions remained stable from then to the close of 2018. The decreasing number of published items since then will need further investigation in future journal analysis. It reflects on one hand that a significant number of manuscripts, being out of the journals’ scope, have been forwarded to the transfer desk. On the other hand, it shows the increasing quality requirements by the reviewers.

We strongly recommend the authors to use ORCID for your manuscript submissions to provide a unique and persistent digital identifier. With your personal ORCID iD authors can link your professional information, affiliations, grants, publications, peer review, and more to your research work and increases your visibility in the scientific community.

### Topical collections

Topical collections became an important strategic instrument to promote relevant and new research themes in environmental earth sciences. In general, topical collections are open and researchers are encouraged to contribute to these structured publication projects. The following list is a compilation of the open topical collections and includes the corresponding guest editors.^9^

**Fig. 8:**
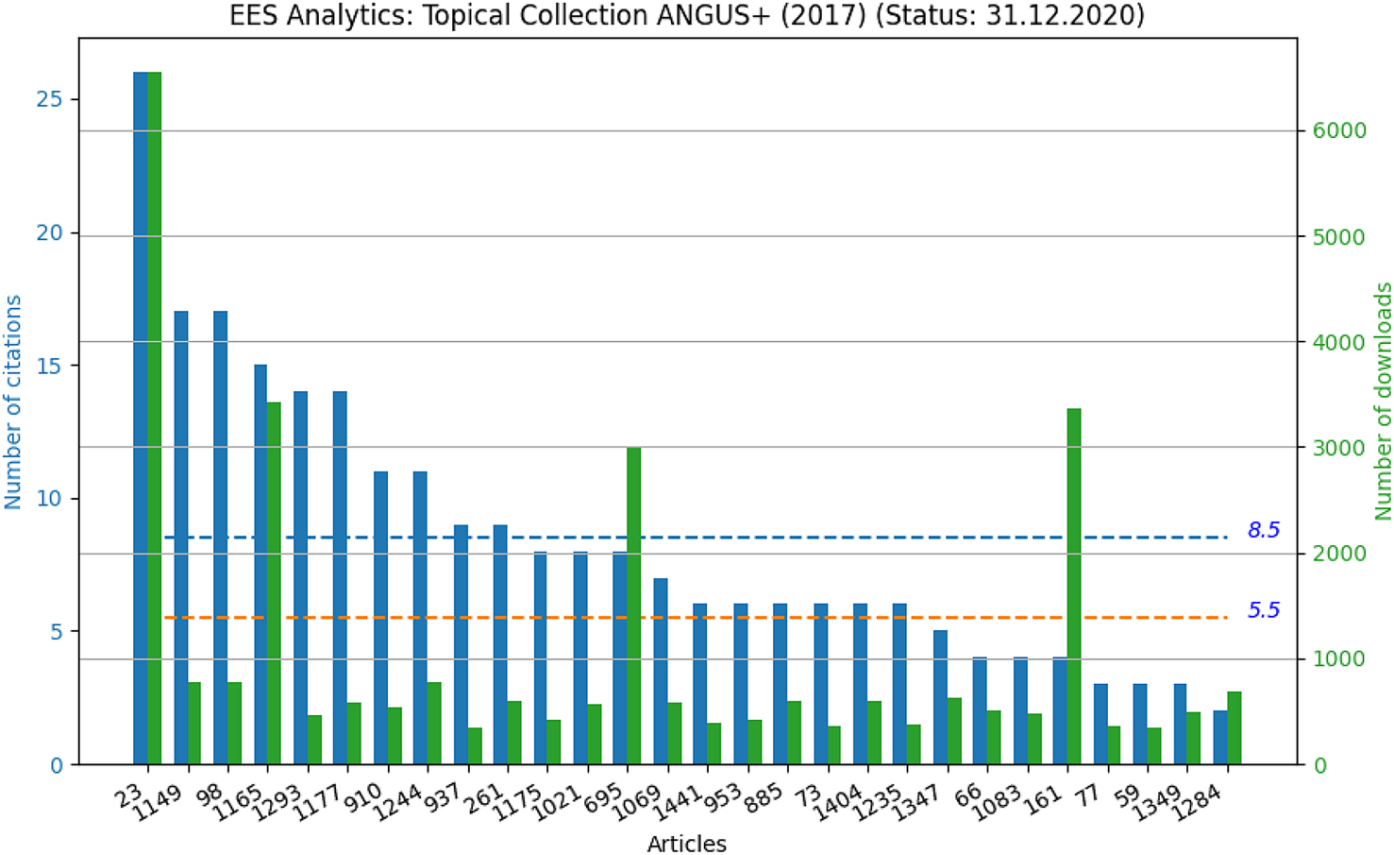
ANGUS Topical Collection on Subsurface Energy Storage (Kabuth et al. 2017)

#### Open topical collections

- Earth Surface Processes and Environment in a Changing World: Sustainability, Climate Change and Society (Alberto Gomes, Horácio García, Alejandro Gomez, Helder I. Chaminé)
- Coastal and Marine Geographic Information System using IoT (Gunasekaran Manogaran, Hassan Qudrat-Ullah, Qin Xin)
- Building stones and geomaterials through history and environments - from quarry to heritage. Insights of the conditioning factors (Siegfried Siegesmund, Luís Sousa, Rubén Alfonso López)
- Groundwater quality and contamination and the application of GIS (Narsimha Adimalla, Hui Qian)
- NovCare - Novel Methods for Subsurface Characterization and Monitoring: From Theory to Practice (Uta Sauer and Peter Dietrich)
- Sustainable Utilization of Geosystems (Ulf Hünken, Peter Dietrich, Olaf Kolditz)
- Visual Data Exploration (Karsten Rink, Roxana Bujack, Stefan Jänicke, and Dirk Zeckzer)
- Geosphere-Anthroposphere Interlinked Dynamics: Geocomputing and New Technologies (Sebastiano Trevisani, Marco Cavalli, Fabio Tosti)
- Global Change of Groundwater in Western Mediterranean Countries (María Luisa Calvache, Carlos Duque, David Pulido-Velazquez)
- Advances in Environmental Geochemistry (Eleanora Carol, Lucia Santucci, Botto Lia)
- Water in Large Basins (Peiyue Li)
- Water Problems in Eastern Mediterranean Countries (H. Gokcekus, D. Orhon, V. Nourani, S. Sozen)

Topical Collections play a central role in a thematic combination of research works, they belong also to the most cited EES papers. Here we continue the detailed analysis for the Topical Collection on “Subsurface Energy Storage” (Kabuth et al. 2017) (Fig. 8) started in Kolditz et al. (2018). The orange line shows a cumulative impact factor of EES (i.e. summing up IFs from 2017 to 2019). The blue line represents the contribution of this collection to the impact factor, i.e. mean value of citations of all related papers for the same period. The reanalysis of the Topical Collection on “Subsurface Energy Storage” indicates an increasing attention of the geoenergy theme.

We are very grateful to the guest editors for organizing and managing these thematic issues of coordinated research works^10^.

## Footnotes

1 Environ Geol (2008) 55:1155-1157 10.1007/s00254-008-1505-8.

2 https://en.wikipedia.org/wiki/Impact_factor.

3 https://webofknowledge.com/.

4 https://www.scopus.com.

5 The name of the journal was changed in 2009 from Environmental Geology to Environmental Earth Sciences; therefore, the analysis under its new name starts a couple of years later.

6 Normally, the number of citations continues to increase a little in the following year due to some late registrations of published items from the previous year.

7 Please note that these are average numbers, specific cases may take a different time particularly when reviewers are difficult to find.

8 Note that the numbers of 2020* are not complete yet due to later registrations. The numbers are as of 31.12.2020.

9 More information can be found at https://www.springer.com/journal/12665/updates/17856126.

10 The entire list of Topical Collections can be found at https://link.springer.com/journal/volumesAndIssues/12665?tabName=topicalCollections.
